# Selective Area Growth and Structural Characterization of GaN Nanostructures on Si(111) Substrates

**Published:** 2018

**Authors:** Alexana Roshko, Matt Brubaker, Paul Blanchard, Todd Harvey, Kris A. Bertness

**Affiliations:** National Institute of Standards and Technology (NIST), Boulder, CO 80305, USA;

**Keywords:** gallium nitride, selective area growth, polarity, defects, PAMBE, TEM

## Abstract

Selective area growth (SAG) of GaN nanowires and nanowalls on Si(111) substrates with AlN and GaN buffer layers grown by plasma-assisted molecular beam epitaxy was studied. For N-polar samples filling of SAG features increased with decreasing lattice mismatch between the SAG and buffer. Defects related to Al–Si eutectic formation were observed in all samples, irrespective of lattice mismatch and buffer layer polarity. Eutectic related defects in the Si surface caused voids in N-polar samples, but not in metal-polar samples. Likewise, inversion domains were present in N-polar, but not metal-polar samples. The morphology of Ga-polar GaN SAG on nitride buffered Si(111) was similar to that of homoepitaxial GaN SAG.

## 1. Introduction

Selective area growth of GaN nanowires (NWs) is a promising approach for obtaining defect-free NW arrays with microstructural, compositional, and spatial uniformity as well as precise position control [1–4]. It is, however, a complex process with conflicting requirements. Successful SAG requires sufficient nucleation to completely fill mask openings, while at the same time nucleation on the mask must be suppressed. Similarly, lateral growth is necessary to fill mask openings, yet conditions for this can be substantially different from those for vertical NW growth. In addition, defects such as dislocations, inversion domains, and stacking faults, must be excluded to achieve optimum performance.

A substantial body of work has been performed on SAG of GaN nanowires. Several growth methods, including metal-organic chemical vapor deposition (MOCVD), plasma-assisted molecular beam epitaxy (PAMBE), and hydride vapor phase epitaxy, have been used on Al_2_O_3_ and Si(111) substrates, with and without AlN and GaN buffers, and with a variety of mask materials. For many applications the conductivity of Si and its prevalence in established technologies make it a preferred substrate.

We have examined the selective area growth of GaN nanostructures by PAMBE on Si(111) substrates with various nitride buffer layers. For hole sizes up to 300 nm and appropriately chosen growth conditions, the degree to which patterned apertures were filled varied with the lattice mismatch between GaN and the buffer on which the NWs were grown. SAG of larger features on lattice matched substrates was limited by defects in the buffers. Hillocks and holes were found at the Si(111)/AlN(0001) interface and these defects propagated to form voids in N-polar GaN buffer layers. While similar defects were found on the Si(111) surface for metal polar nitride growth, they did not cause voids in the Ga-polar buffer. Likewise, inversion domains were found in the N-polar but not the Ga-polar buffers. Ga-polar SAG nanostructures on nitride buffered Si(111) had morphologies similar to homoepitaxial GaN SAG on Ga-polar GaN.

## 2. Materials and Methods

### 2.1. Growth

The samples studied were grown by plasma-assisted molecular beam epitaxy. Growths were on Si(111) substrates and on a commercial N-polar GaN substrate (Kyma Technologies, Raleigh, NC USA). The material vendor is identified only to adequately specify the material. It does not imply recommendation or endorsement by the National Institute of Standards and Technology, nor does it imply that the product identified is necessarily the best available for the purpose. Details of the N-polar and Ga-polar buffer growths and SAG, including temperatures and atomic fluxes, have been described previously [5,6]. Three different types of N-polar buffers were grown on the Si(111) substrates: AlN, GaN on AlN, and GaN with a GaN/AlGaN superlattice on AlN. The GaN buffers with GaN/AlGaN superlattices were ~7 times thicker than the GaN buffer with no superlattice. A fourth buffer type was grown with metal polarity: GaN with a GaN/AlGaN superlattice on AlN.

Nanowires were grown under conditions that produced full selectivity, with the exception of the growth on the commercial N-polar GaN which was lower in temperature due to reduced heater coupling to this transparent substrate. The selective area growths were on chemical vapor deposited SiNx masks patterned by e-beam lithography [5]. After lithography and prior to SAG the samples were cleaned with an O_2_ reactive ion etch to remove organics followed by HCl cleaning to remove oxygen and other inorganics [7]. The e-beam holes studied were 300 nm in diameter. E-beam lines, 5 μm by 100 to 300 nm, were also examined.

Several samples of each buffer type were prepared. In some cases, different measurements were made on different samples, but the samples were prepared under nominally identical conditions. The polarity of the buffers and SAG nanostructures were confirmed for each sample type by scanning transmission electron microscopy annular bright field imaging (described below). The different buffer types and thicknesses of each layer are reported for the N-polar samples in Table 1

### 2.2. Strain Analysis

The lattice parameters of the N-polar GaN buffer layers were assessed by X-ray diffraction (XRD). Measurements were made with a double crystal diffractometer equipped with a Cu Kα source (λ = 0.15418 nm), a Ge(220) hybrid monochromator, and a Ge(220) triple axis receiving optic. Each peak was recorded separately. Bragg peak positions were determined by parabolic fitting of omega-2theta scans. The c-lattice parameters were calculated from the symmetric (0002) (0004) (0006) peaks and then used to determine a-lattice parameters from the asymmetric (10–14) (10–15) and (20–24) peaks [8]. The a-lattice parameters and their errors, reported in Table 1, are the average of the values determined from the three asymmetric peaks and their standard deviation respectively.

### 2.3. Morphology Analysis

Sample morphology was characterized by field emission scanning electron microscopy (FESEM). Images were taken at 5 kV with an in-lens detector in both plane-view and tilted (incident beam 20 ° from the specimen surface normal) orientations.

High resolution images of the sample polarity and defect structure were taken with a field emission, scanning transmission electron microscope (STEM) equipped with a spherical aberration probe corrector. Images were acquired at 200 kV with both annular bright field (ABF) and high angle annular dark field (HAADF) detectors. The ABF images, of the Ga and N atomic columns, were recorded with an illumination half angle of 22.7 mrad, and inner and outer ABF detector collection half angles of 11 and 22.5 mrad respectively. Images were taken along the $\left[11\bar{2}0\right]$ zone axis for all samples except N-AlGa, which was imaged along the $\left[1\bar{1}00\right]$ zone axis. All STEM images are single-frame, unprocessed images.

Lamellae for STEM examination were prepared by focused ion beam (FIB) thinning. A protective Pt layer was deposited on the region of interest prior to thinning, then a Ga-ion beam was used to mill out an electron transparent section. Coarse milling (down to ~1 um lamella thickness) was carried out in steps of 30 kV:16 nA, 30 kV:4 nA, and 30 kV:1 nA. The sample was then milled to electron transparency at 30 kV:50 pA followed by 5 kV:20 pA. For some samples FIB damage was removed by subsequent Ar-ion milling at 900 and 500 eV at 150 μA and ± 10 °. Other samples were FIB milled for longer periods with a 5 kV:20 pA beam, at ±3 degrees, with short beam dwell times; this lower energy FIB milling sufficiently removed damage so that Ar-ion milling was not required.

## 3. Results

The in-plane, nitride lattice parameters, a_o_, determined from X-ray diffraction measurements of the three N-polar buffer types are shown in Table 1. Also reported is the lattice mismatch expressed as a percentage, Δa_o_ (%), for relaxed GaN, a_o_ = 0.3189 nm [9], relative to the in-plane lattice parameter of each buffer:

(1) $${\Delta \text{a}}_{\text{o}}(\text{\%})=100\times \left[{\text{a}}_{\text{o}}(\text{GaN})-{\text{a}}_{\text{o}}(\text{buffer})\right]/{\text{a}}_{\text{o}}(\text{ buffer }).$$

For the AlN only buffer (N–Al) the in-plane lattice parameter measured was 0.3115 ± 0.0001 nm. This is in good agreement with published values for AlN, a_o_(AlN) = 0.3112 nm [10], suggesting the AlN buffer is nearly completely relaxed [11–15], and leading to a large mismatch, 2.4%, between the AlN buffer and GaN in-plane lattice parameters. In specimen N-AlGa, the addition of a thin (45 nm) GaN buffer layer, on top of the AlN buffer, reduced the lattice mismatch for the SAG GaN by an order of magnitude of ~0.2%. Increasing the GaN buffer thickness further, to ~300 nm, and adding an AlGaN/GaN superlattice (specimens N-AlGaSL-1 and N-AlGaSL-2) reduced the in-plane lattice mismatch nearly another order of magnitude, to less than 0.02%. The commercial N-polar GaN substrate used (specimen N-GaN) was assumed to be fully relaxed with no mismatch for GaN SAG.

**Figure 1.**
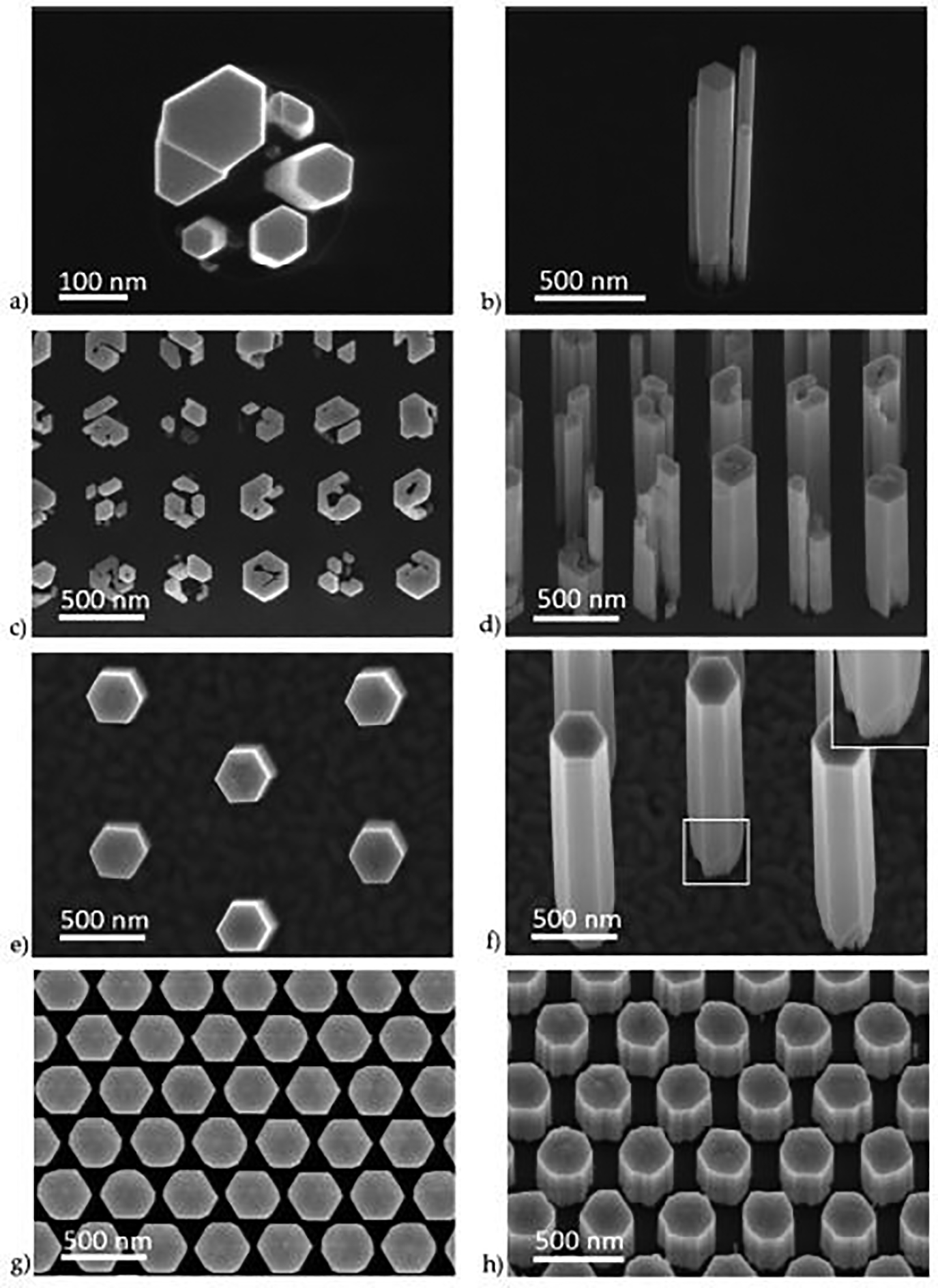
Plane-view field emission scanning electron microscopy (FESEM) images of selective area growth (SAG) nanowires on different buffers: (**a**) and (**b**) N–Al (AlN/Si(111) substrate; Δa_o_ = 2.369%), (**c**) and (**d**) N-AlGa (N-polar GaN/AlN/Si(111) substrate; Δao = −0.213%), (**e**) and (**f**) N-AlGaSL-1 (N-polar GaN + SL/AlN/Si(111) substrate; Δa_o_ = 0.028%), (**g**) and (**h**) N–GaN (commercial N-polar GaN substrate. The diameters of the patterned holes were 300 nm for all images.

The influence of the different in-plane lattice mismatches on the selective area growth of GaN NWs can be seen in Figure 1. The patterned hole size after lithography was 300 nm for all images. For SAG nanowires grown on the AlN buffer (Figure 1a,b, specimen N–Al) the large in-plane lattice mismatch resulted in multiple GaN nucleation sites and incomplete filling of the holes. On this sample, the e-beam pattern had only widely spaced holes (≥5 μm) so only one hole is shown rather than an array. Figure 1a,b are of holes with some of the highest fill factors on this sample, yet the holes are still only partially filled. It can also be seen that while some of the nuclei grew together, most did not, because strain caused by the very large lattice mismatch limited the lateral growth of the nuclei. In other words, strain, which aids self-assembly of GaN nanowires by limiting lateral growth [16–21], detracts from selective area NW growth. Reducing the in-plane mismatch by an order of magnitude, from 2.3% for sample N–Al to 0.2% for sample N–AlGa, increased the hole fill-factor, as can be seen in Figure 1c,d. However, there were still multiple nucleation sites in the apertures on this lower misfit sample and the apertures remain incompletely filled. As can be seen in Figure 1c, nucleation on this sample occurred predominantly at the edges of the holes.

Preferential nucleation at mask edges was observed in many samples (not shown here). In an earlier study of GaN SAG on GaN-on-sapphire templates, a kinetic model was proposed which attributed edge nucleation to a weaker interaction of Ga adatoms with the mask than with the GaN template, causing Ga adatoms to become “trapped” at the borders of the nanoholes [22]. In addition surface energy is expected to promote edge nucleation, since step and edge nucleation are well-known to minimize surface energy [23,24]. The previously proposed model for GaN SAG also described the coalescence of GaN into a single nanocrystal prior to the formation of a hexagonal NW shape and prior to significant vertical growth [22]. This differs from the observation in this study, that uncoalesced nuclei persist at the base of both incompletely (Figure 1b,d) and completely (Figure 1f) filled nanoholes, even after substantial vertical growth. This difference is likely due to the different lattice mismatch and sample polarity in the two studies. For the previous work, strain should not limit the lateral growth of nuclei on the GaN template, and faster diffusion of N adatoms on the Ga-polar surface, relative to that on the N-polar surfaces used in this study [25] should also increase nuclei coalescence.

Because the conditions for N-polar and Ga-polar film and NW growth are substantially different [6,26] the conditions for selective area growth of the two polarities are also expected to be different. Accurate identification of growth polarity is, therefore, necessary for understanding the SAG process. Unfortunately, there has been ongoing confusion regarding crystallographic polarity for GaN NWs [27]. Many early determinations of GaN polarity were by convergent beam electron diffraction (CBED), which requires correct measurement of the rotation angle between the diffraction pattern and the image, as well as simulations of the CBED patterns. In a recent study of GaN polarity, rotations of 180° and 120° between the CBED pattern and sample image were reported [27]. If unaccounted for, these rotations would cause incorrect polarity assignment. The advent of STEM ABF imaging has enabled direct determination of nitride polarity from atomic scale images of N and metal columns [28]. In addition, the relationship between nanowire polarity and tip geometry has been established for many growth conditions [29,30], although, the presence of inversion domains (IDs) can complicate morphology based polarity identification [29,31].

While the N-polar NWs studied here did not all coalesce completely before vertical growth began, their behavior generally followed the model that each SAG nanowire is formed from a collection of nuclei which must grow together. On buffers with large mismatch, the higher interfacial energy due to mismatch strain limits or prevents lateral expansion of the nuclei on the buffer. As the mismatch and corresponding interfacial energy are reduced, the size of an unstrained nucleus increases until eventually layer by layer growth can be achieved [32]. This is one advantage of homoepitaxial SAG, and it explains why reducing the in-plane lattice mismatch another order of magnitude (from 0.2% to 0.02%) resulted in completely filled e-beam holes for diameters up to 300 nm. Complete aperture filling for specimens N–AlGaSL-1 and N–GaN, is shown in Figure 1e–h, indicating there was no barrier to the lateral growth of GaN nuclei on the lattice-matched buffer and substrate.

**Figure 2.**
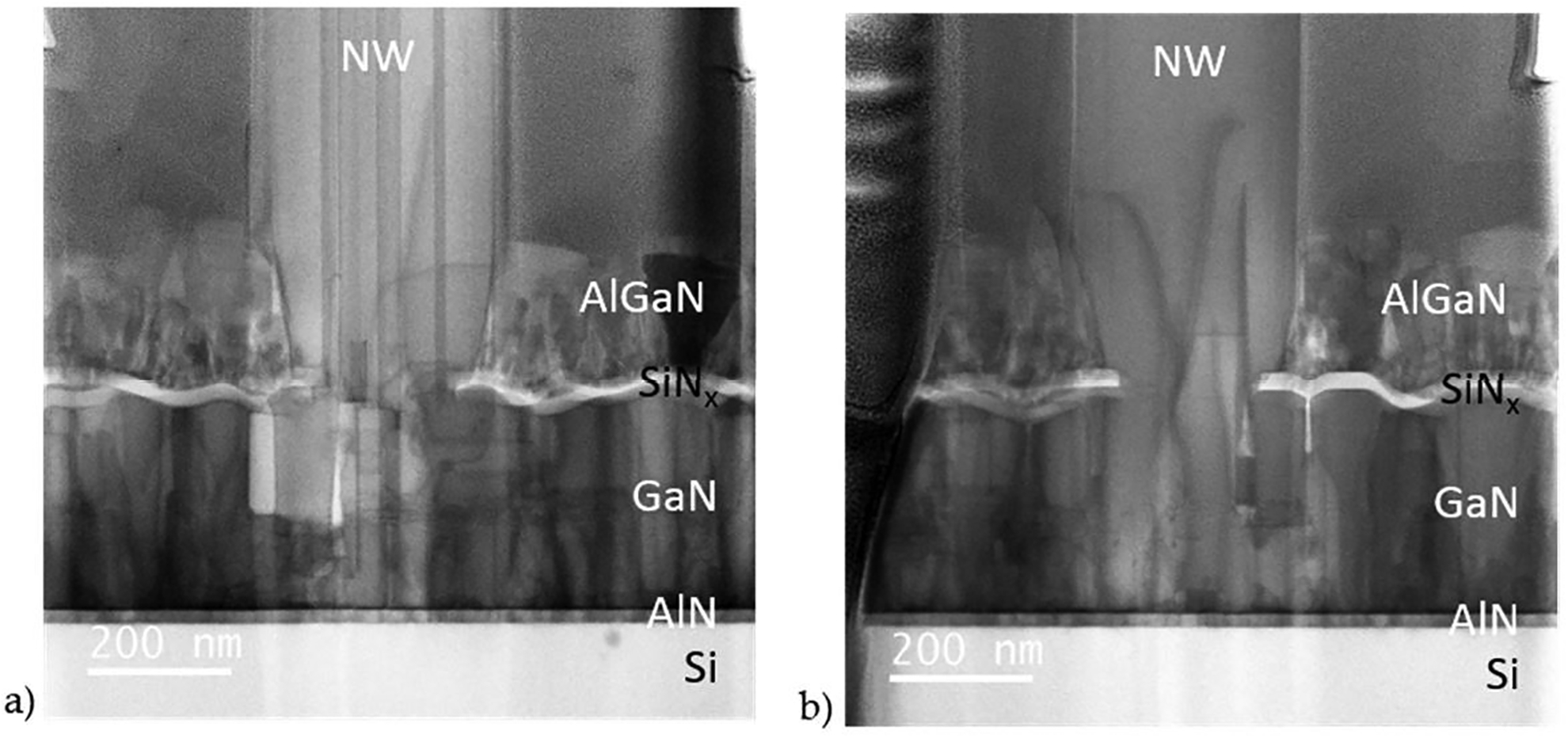
STEM ABF images of NWs on sample N–AlGaSL-1 showing: (**a**) inversion domains, some of which propagate from the GaN buffer and some of which initiate at the regrowth interface; (**b**) threading dislocations propagating from the GaN buffer into the NW and annihilating near the NW base. After the NW growth, an AlGaN layer was deposited on this sample at low a temperature and can be seen on the SiNx buffer in both images.

It is interesting that complete aperture filling was achieved for sample N–AlGaSL–1 despite defects in the underlying buffer, including voids, threading dislocations (TDs) and inversion domains (IDs). As shown in Figure 2, many of these defects propagated from the buffer into the SAG NWs. In addition to IDs propagating from the buffer, Figure 2a shows IDs that start at the regrowth interface. ID formation at GaN SAG regrowth interfaces has been associated with Al, O, and Ti contamination [7,33]. IDs can also form on localized stacking faults [33], at steps in the interface [34], and on pinholes in the buffer [35]. It is possible that some or all of these formation mechanisms occurred in sample N-AlGaSL-1. Of the four NWs imaged from this sample two had IDs and two had none. IDs were observed in all of the SAG samples shown in Figure 1, however, not every SAG NW within each sample contained IDs. Similarly, for all samples threading dislocations were observed in some but not every NW imaged. When they did occur, TDs annihilated near the bottom of the NW by bending to the surface, consistent with reports of “dislocation filtering” [2,3,31,36].

**Figure 3.**
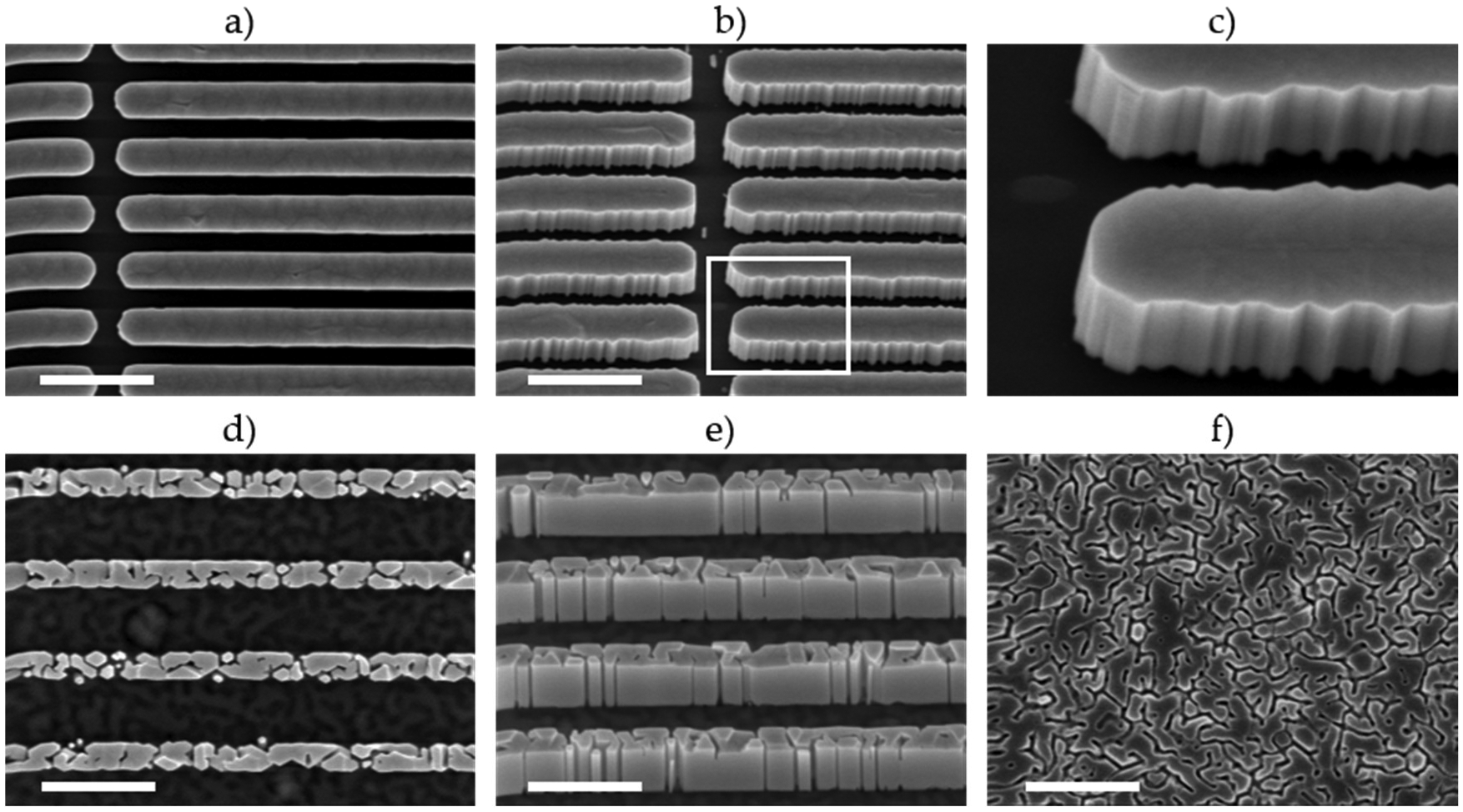
FESEM images of SAG lines: (**a**), (**b**), and (**c**) on a commercial N-polar GaN substrate (N-GaN), 300 nm wide; (**d**) and (**e**), on a PAMBE grown N-polar GaN + SL/AlN/Si(111) substrate (N–AlGaSl-2), 250 nm wide. Complete filling of the lines is achieved on the N-polar GaN substrate, but not on the PAMBE substrate. Image (**f**) is of a bare PAMBE grown N-polar GaN + SL/AlN/Si(111), taken prior to processing for SAG. The scale of all the images is the same (white lines are 1 μm long), except (**c**) which is a magnified view of the area outlined in (**b**) and shows m-plane faceting of sidewalls patterned along the a-plane.

Holes with diameters larger than 300 nm were not patterned on these samples, however, lines 300 nm and 250 nm by 5 μm were patterned respectively on the commercially grown N-polar GaN substrate (N–GaN) and a GaN/AlN superlattice buffer (N-lGaSL-2, Δa_o_ = 0.006%) (Figure 3). As expected for true homoepitaxial growth, the SAG nanowalls on the commercial N-polar GaN substrate were completely filled (Figure 3a–c). The difference in sidewall roughness of the two sets of nanowalls shown for this sample resulted from the lines being patterned along orthogonal directions. Nanowalls patterned along the $\left\{10\bar{1}0\right\}$ planes (i.e., the $<11\bar{2}0>$ directions) of the underlying GaN crystal have smooth m-plane sidewall facets (Figure 3a). Nanowalls patterned along the $\left\{11\bar{2}0\right\}$ planes (the $<10\bar{1}0>$ directions), which would ostensibly lead to a-plane sidewalls, faceted to form lower energy m-plane $\left\{10\bar{1}0\right\}$ surfaces, as shown in Figure 3b and at higher magnification in Figure 3c. This is consistent with theoretical calculations, which report slightly lower surface energy for the m-plane than for the a-plane [37–40].

**Figure 4.**
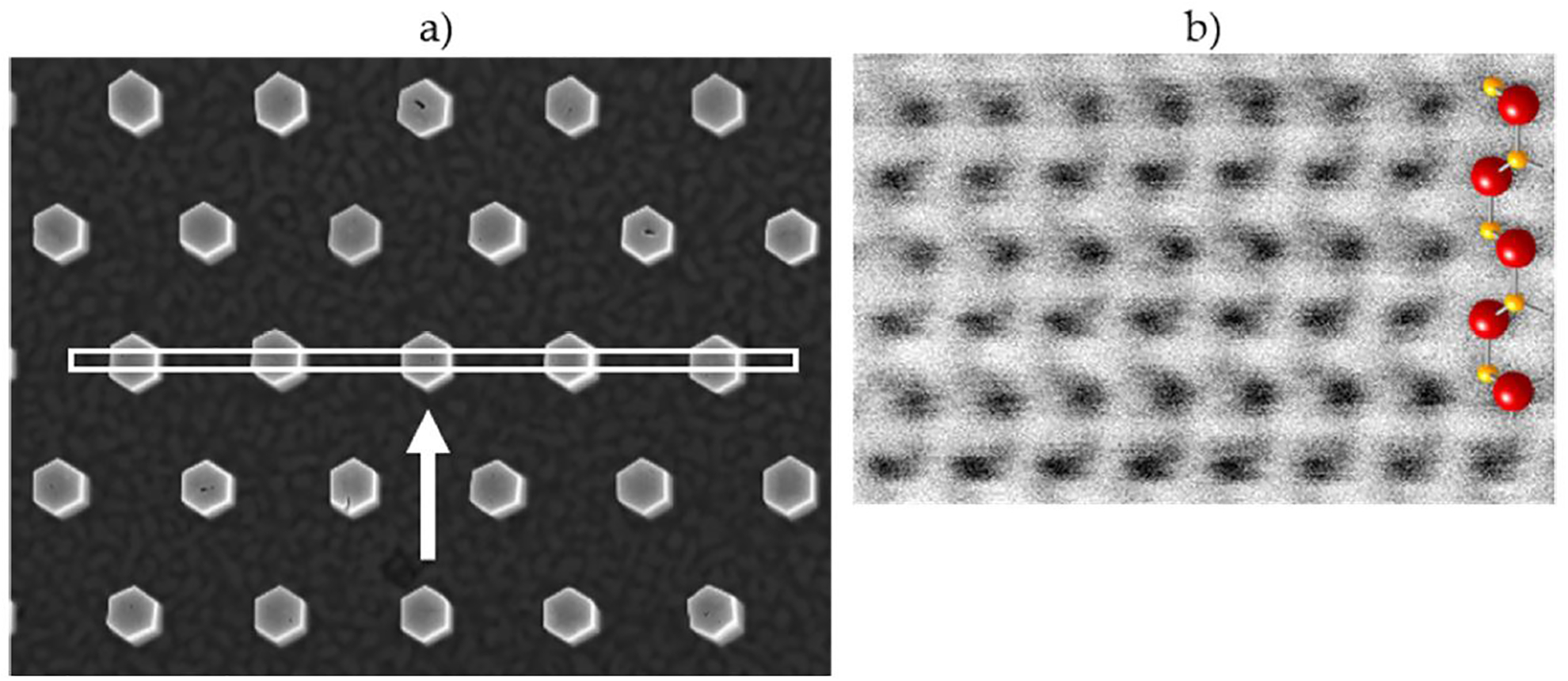
(**a**) Schematic of the STEM lamellae position on sample N–AlGaSL-1; the arrow indicates the STEM imaging direction. (**b**) Atomic resolution STEM ABF image showing the zone axis is $\left[11\bar{2}0\right]$ and, therefore, lamella has $\left\{11\bar{2}0\right\}$ surfaces and the NW facets are $\;[1\bar{1}00]$ The atomic resolution image also shows the nitrogen polarity of the NWs.

Preferential formation of m-plane facets has also been reported for self-assembled GaN NWs [41,42], for Ga-polar SAG GaN nanowires grown by PAMBE [22], and it was observed for the N-polar SAG NWs in this study. Although the patterned apertures on all samples were round, as the NWs grew vertically they also expanded laterally to form m-plane sidewall facets (see Figure 1f). STEM imaging was used to verify the facet planes. As shown in Figure 4a, lamellae were prepared with the imaging plane perpendicular to a pair of sidewall facets. ABF imaging showed the zone axis was $\left[11\bar{2}0\right]$ (Figure 4b), indicating the lamella had $\left\{11\bar{2}0\right\}$, or a-plane surfaces, and confirming the NW facets are $\left\{10\bar{1}0\right\}$ or m-planes. The ABF image, (Figure 4b), also shows the NW is N-polar. M-plane surface facets and N-polarity were found for all samples in Figure 1 except the commercial N-polar substrate (N–GaN), which had a mix of m- and a-plane facets (Figure 1g,h) resulting from the lower growth temperature for SAG on this GaN only substrate (see “Growth” description above). Growth on this sample was also N-polar.

Surprisingly the SAG nanowalls on the GaN/AIN buffer with virtually no lattice mismatch, 0.006% (N–AlGaSl-2), did not fill completely (Figure 3d,e). This incomplete filling cannot be attributed to the SAG conditions, which were similar to those used for the completely filled SAG nanowalls on the N- polar GaN substrate (N–GaN, Figure 3a,b). In fact, the GaN regrowth on N–AlGaSL-2 was longer than that on N–GaN, as can be seen from the difference in the nanowall heights (compare Figure 3b,e), which might be expected to lead to more complete filling. Instead, the SAG nanowalls on N–AlGaSL-2 have a granular structure with a relatively large void fraction. A similar granularity is evident in the surface topography of the underlying buffer, visible between the nanowalls in Figure 3d and in a nominally identical GaN buffer, before deposition and patterning of the SiNx mask, (Figure 3f, contrast in this image was increased to highlight the granularity). This suggests the granularity of the SAG nanowalls is replicating that of the underlying buffer surface. The size of the granularity in these two images was measured using a line intercept method. Over an aggregate line length of 33 μm for each sample, the average size of the “grains” was determined to be 254 nm for the SAG nanowalls and 227 nm for tire buffer surface. The 12% larger “grain” size determined for the nanowalls compared with that of the buffer is likely due to lateral growth and/or coalescence of “grain” within the nanowalls during; growth and/ or to small unintentional differences between buffer runs, consistent with the buffer granularity causing the; incomplete line filling.

The granular structure of the GaN buffer is similar to that observed for GaN layers geown directly on Si(111), ([43], Figure 3a; [4] Figure 1a). In one study, the granularity was attributed to surface roughness induced by evaporation of excess Ga atoms and to thermal decomposition of GaN at dislocations [43]. In the other study, high-resolution TEM imaging showed the presence of a thin amorphous SiNx layer at the GaN/Si(111) interface on which GaN islands formed with slightly different orientations, leading to a mosaic, granular surface structure [4].

**Figure 5.**
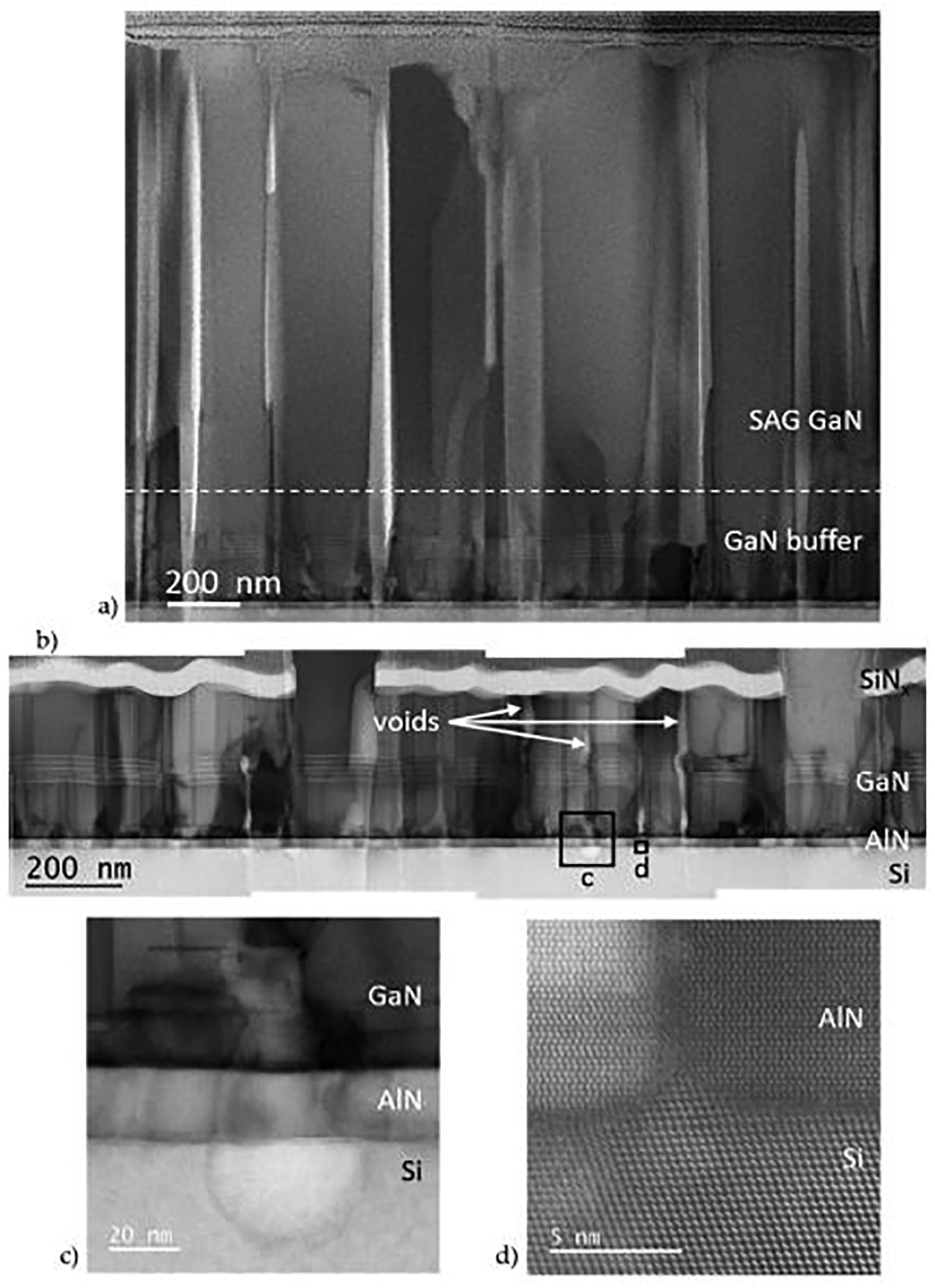
(**a**) through (**c**) STEM ABF images of the N–AlGaSL-2 buffer: (**a**) lower magnification image showing the columnar structure associated with the granularity: observed in the SEM (Figure 2d,e), the dashed white line marks the interface between the GaN buffer and the SAG GaN; (**b**) lower magnification composite image showing columnar voids in the GaN buffer beneath the SiNx mask; (**c**) high magnification image of a void which initiates in the Si buffer (left box in (**b**)); and (**d**) atomic scale STEM HAADF image at the base of another void (right box in (**b**)) where the Si protrudes above the substrate plane and generates a defect in the AlN buffer.

STEM imaging was used to investigate the source of the buffer and SAG granularity in this study. As can be seen in Figure 5a, the voids in N-AlGaSL-2 extend down through the SAG GaN to the GaN buffer. This is also visible in the tilt view SEM image of this sample, Figure 3e, which shows holes in the SAG GaN nanowall extend vertically down to the buffer surface, with straight edges. In Figure 5a, many of the voids appear to propagate through the GaN buffer layer. Because this could be an artifact of the FIB milling process, we examined the buffer layer underneath the SiNx mask, which should protect it during the lamella preparation. Voids in the GaN buffer are clearly visible even beneath the SiNx (Figure 5b) indicating they are formed during buffer growth and are not artifacts. Unlike voids in the SAG GaN, which have very straight sidewalls, the width and sidewall angles of the voids in the GaN buffer vary along their length.

Most of the voids in the GaN layer appear to originate in the AlN buffer and in many cases at defects in the Si(111)/AlN interface. In several instances, voids were observed in the Si substrate surface; these voids persisted through the buffer layers (Figure 5c). Voids in the buffer were also found to initiate at Si protrusions above the Si(111)/AlN interface. Figure 5d is a STEM HAADF image which shows the Si(110) dumbbells extend above the plane of the Si(111) surface into the AlN layer. This Si hillock causes a defect in the AlN which becomes a void in the GaN buffer, visible in Figure 5b.

Defects in the Si(111)/AlN interface were observed in all of the samples studied, and are likely related to the low temperature at which the Si–Al eutectic forms (577 °C) [44] relative to the PAMBE growth temperatures (785 and 675 °C for the AlN and GaN buffers respectively). Because Al is not soluble in Si in the solid phase, no second phase formation is expected. But compositional nonuniformities in the growth interface at the beginning of growth could cause localized surface melting due to eutectic formation, resulting in the holes and hillocks observed on the Si surface.

**Figure 6.**
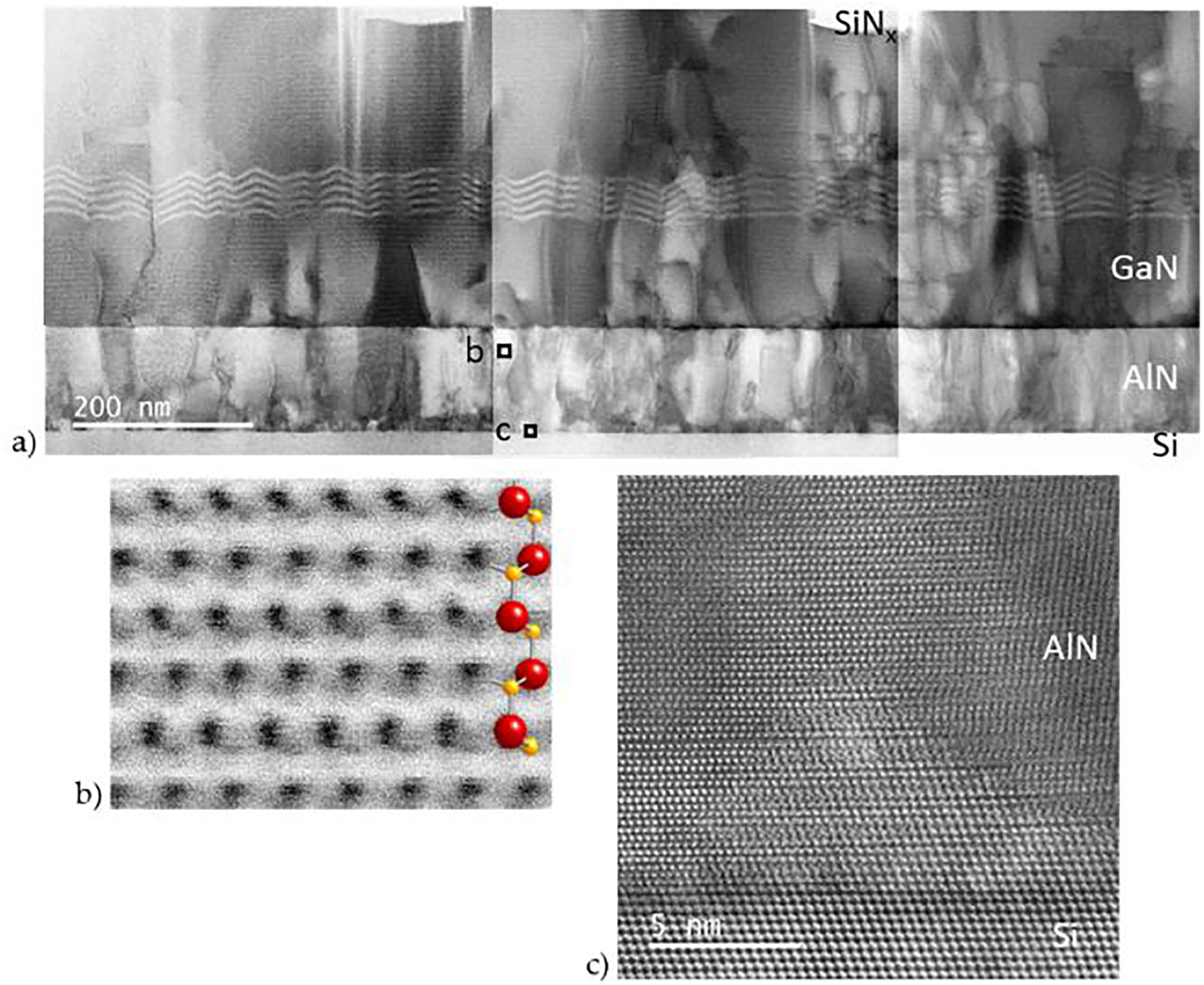
(**a**) and (**b**) STEM ABF images of the Ga-polar buffer for Ga–AlGaSL: (**a**) lower magnification composite image showing threading dislocations and mosaic structure in the Al-polar AlN, which propagate into the GaN buffer; (**b**) atomic resolution STEM ABF image showing Al-polarity in the AlN buffer (top box in (**a**)); and (**c**) atomic scale STEM HAADF image of an Si hillock above the substrate plane at the Si/AlN interface (bottom box in (**a**)), similar to that in Figure 4d.

Ga-polar PAMBE GaN has been reported to have smoother surface morphologies than N-polar GaN [43]. Therefore, an additional sample was grown with metal polarity (Ga–AlGaSL). The sample polarity was confirmed by STEM ABF imaging, Figure 6b. As can be seen in the lower magnification composite STEM ABF image of this sample, Figure 6a, the AlN buffer has a mosaic structure and numerous threading dislocations, most of which continue into the GaN buffer. In addition, Si hillocks were observed on the Si(111) surface, Figure 6c, similar to those found in the N-polar samples. In contrast to the N-polar samples, however, defects in the Si surface did not persist to initiate voids in the GaN layer, Figure 6a. The lack of cavities in the Ga-polar GaN may be due to enhanced N-adatom mobility on the Ga-polar GaN surface [25,45–47]. There may also be a lower density of eutectic voids at the Si interface in this sample, since it was grown with a much lower Al-flux. Unfortunately, the relatively small area analyzed by STEM does not provide sufficient statistics to confirm this hypothesis. Another noteworthy distinction between this and the N-polar samples, is that no inversion domains were observed in the Ga-polar GaN (compare Figures 6a and 5b). An absence of IDs has been reported previously for Ga-polar films and SAG NWs grown by PAMBE [30,48], and for Ga-polar films grown by MOCVD [49–51], and is expected to be advantageous for Ga-polar devices. FESEM images (not shown here) of SAG nanowalls on this sample, 300 nm by 5 μm, show complete filling with uniformity roughly equivalent to SAG on the commercial N-polar substrate, N–GaN, (Figure 3a,b).

## 4. Conclusions

In summary, we have shown that decreasing the lattice mismatch between GaN and N-polar buffer layers increases the fill factor of SAG NWs with diameters up to 300 nm. We have also observed the “dislocation filtering” effect, wherein very few threading dislocations propagate into these 300 nm diameter NWs and those that do annihilate near the NW base. Defects in the surfaces of the Si(111) substrates were found in all of the samples and are probably caused by localized eutectic formation at the beginning of tine AlN buffer growth. For N-polar samples these defects persisted through the buffers and caused voids in SAG nanowalls, but not in SAG nanowires. These defects did not propagate in metal polar buffers, possibly due to enhanced N diffusion on the Ga-polar surface, and SAG nanostructures on this buffer were similar to homoepitaxial GaN SAG.

## Figures and Tables

**Table 1.** Structure and in-plane lattice mismatch for the N-polar buffers studied.

Sample^1^	Substrate/Buffer	AlN (nm)	GaN Buffer (nm)	SiNx (nm)	Buffer a_o_^2^ (nm)	Δa_o_ mismatch (%)^3^
**N-Al**	Si(111)/AlN	40 ± 5	0	74	0.3115 ± 0.0001	2.4 ± 0.2
**N-AlGa**	Si(111)/AlN/GaN	50 ± 5	45 ± 5	54	0.3195 ± 0.0005	−0.18 ± 0.06
**N-AlGaSL-1**	Si(111)/AlN/GaN + SL	20 ± 2	295 ± 15	25	0.3188 ± 0.0002	0.019 ± 0.002
**N-AlGaSL-2**	Si(111)/AlN/GaN + SL	20 ± 2	305 ± 20	50	0.3189 ± 0.0001	−0.006 ± 0.0004
**N-GaN**	N-polar GaN	0	0	50	0.3189^4^	0

1 Sample polarity was verified by STEM ABF imaging.

2 Calculated by fitting (0002) (0004) (0006) (10–14) (10–15) and (20–24) XRD Bragg peaks; the error is the standard deviation of the a_o_ values determined from the three asymmetric peaks.

3 Mismatch was calculated as described in the text, assuming a relaxed GaN a_o_ of 0.3189 nm.

4 Assumed, not measured.
